# An improved digital polymerase chain reaction protocol to capture low‐copy *KRAS* mutations in plasma cell‐free DNA by resolving ‘subsampling’ issues

**DOI:** 10.1002/1878-0261.12110

**Published:** 2017-08-08

**Authors:** Yusuke Ono, Ayumu Sugitani, Hidenori Karasaki, Munehiko Ogata, Reo Nozaki, Junpei Sasajima, Tomoki Yokochi, Shingo Asahara, Kazuya Koizumi, Kiyohiro Ando, Katsunori Hironaka, Tsutomu Daito, Yusuke Mizukami

**Affiliations:** ^1^ Institute of Biomedical Research Sapporo Higashi Tokushukai Hospital Hokkaido Japan; ^2^ Department of Medicine Asahikawa Medical University Japan; ^3^ Department of Clinical Research Chiba Tokushukai Hospital Japan; ^4^ Center for Clinical and Translational Science Shonan Kamakura General Hospital Kanagawa Japan; ^5^ Bio‐Rad Laboratories Co., Ltd. Tokyo Japan

**Keywords:** cancer, cell‐free DNA, droplet digital polymerase chain reaction, liquid biopsy, minimally invasive diagnostics

## Abstract

Genetic alterations responsible for the initiation of cancer may serve as immediate biomarkers for early diagnosis. Plasma levels of cell‐free DNA (cfDNA) in patients with cancer are higher than those in healthy individuals; however, the major technical challenge for the widespread implementation of cfDNA genotyping as a diagnostic tool is the insufficient sensitivity and specificity of detecting early‐stage tumors that shed low amounts of cfDNA. To establish a protocol for ultrasensitive droplet digital polymerase chain reaction (ddPCR) for quantification of low‐frequency alleles within a limited cfDNA pool, two‐step multiplex ddPCR targeting eight clinically relevant mutant *KRAS* variants was examined. Plasma samples from patients with colorectal (*n* = 10) and pancreatic cancer (*n* = 9) were evaluated, and cfDNA from healthy volunteers (*n* = 50) was utilized to calculate reference intervals. Limited cfDNA yields in patients with resectable colorectal and pancreatic cancers did not meet the requirement for efficient capture and quantification of rate mutant alleles by ddPCR. Eight preamplification cycles followed by a second‐run ddPCR were sufficient to obtain approximately 5000–10 000 amplified copies per ng of cfDNA, resolving the subsampling issue. Furthermore, the signal‐to‐noise ratio for rare mutant alleles against the extensive background presented by the wild‐type allele was significantly enhanced. The cutoff limit of reference intervals for mutant *KRAS* was determined to be ~ 0.09% based on samples from healthy individuals. The modification introduced in the ddPCR protocol facilitated the quantification of low‐copy alleles carrying driver mutations, such as oncogenic *KRAS*, in localized and early‐stage cancers using small blood volumes, thus offering a minimally invasive modality for timely diagnosis.

**Abbreviations**
cfDNA: cell‐free DNA
CRC: colorectal cancer
ddPCR: droplet digital polymerase chain reaction
FAM: 6‐fluorescein amidite
FFPE: formalin‐fixed paraffin‐embedded
HEX: hexachloro‐fluorescein
LNA: locked nucleic acid
PDA: pancreatic ductal adenocarcinoma

## 1 Introduction

Identification of novel noninvasive biomarkers would provide a more effective and patient‐friendly tool for the detection of cancer. In this respect, circulating cell‐free DNA (cfDNA) may be a most promising target. cfDNA represents fragments of DNA shed from tumors into the general circulation and has been intensively studied to systematically trace the genomic evolution of cancer (Crowley *et al*., 2013). Hence, plasma cfDNA may be an alternative to cellular DNA obtained by tissue biopsies for cancer diagnostics. Given recent advances in sequencing technology, mutation profiles and copy number alterations for a large number of cancer‐related genes can be assessed in blood and urine specimens instead of primary and/or metastatic tumor tissues acquired through invasive procedures (Forshew *et al*., 2012; Sausen *et al*., 2015). Such an approach may overcome the problems associated with intratumor or intralesion genetic heterogeneity as cfDNA genotyping can cover the whole genetic landscape of cancer in all its complexity (De Mattos‐Arruda *et al*., 2014).

In order to establish an efficient strategy for liquid biopsy in cancer diagnostics, the development of more accurate, reliable, and cost‐effective tools to identify informative mutations is essential. As cfDNA sequencing is still expensive, time‐consuming, and labor‐intensive, its value in clinical practice is limited. Therefore, we focused on digital polymerase chain reaction (PCR) as a simple and low‐cost method to detect tumor‐derived cfDNA. This technology is based on measuring absolute quantities of nucleic acids encapsulated within ‘water‐in‐oil droplet’ partitions, resulting in a detection limit of approximately 0.05–0.01% for the quantification of point mutations (Hindson *et al*., 2011; Taly *et al*., 2013). Consequently, current commercially available platforms demonstrate sufficient sensitivity to distinguish between mutant and wild‐type DNA fragments extracted from patients with advanced tumors.

cfDNA detected in plasma is derived from either cells of normal tissues or mutant cells of the tumor. In general, the amount of cfDNA in plasma is correlated with the tumor burden. However, cfDNA copy numbers can vary among individuals with different tumor types, and the cfDNA level is significantly influenced by cancer histotype (Bettegowda *et al*., 2014). Notably, similar copy numbers of cfDNA can occasionally be detected in healthy individuals and patients with cancer, particularly during the early disease stages, resulting in insufficient diagnostic power to detect tumor‐derived rare mutant cfDNA. In addition, considerable variations in plasma cfDNA concentrations may be due to the type of sample treatment and quantification technique (Devonshire *et al*., 2014), indicating that the detection limit of cfDNA may depend on the analyzed clinical sample. Although droplet digital PCR (ddPCR) has rather high precision and sensitivity for absolute quantification (0.01%), even at very low target concentrations, there may be an intrinsic error due to ‘subsampling’ (Lievens *et al*., 2016). Such issues potentially cause large variations or errors in quantification, even when using a highly accurate platform. Thus, using ddPCR technology for early cancer diagnosis and risk stratification is still challenging.

Here, we aimed to overcome this subsampling issue caused by limited cfDNA yield and missing targets at very low abundance during compartmentalization in ddPCR‐based liquid biopsy assays and to establish a more reliable framework for digital quantification of rare tumor cell‐derived mutant alleles. Such an approach will allow us to conduct minimally invasive procedures for early cancer diagnosis and for the surveillance of individuals at high risk.

## 2 Materials and methods

### 2.1 Ethics statement

The study protocol was approved by Tokushukai Group Ethical Committee on Human Research.

### 2.2 Patients

As a preliminary study population, we selected patients with newly diagnosed colorectal cancer (CRC) or pancreatic ductal adenocarcinoma (PDA) who underwent curative resection at the participating institutions between 2014 and 2016 (*n* = 10 for CRC, *n* = 9 for PDA; Table 1). Fifty healthy volunteers (age range, 21–63 years; mean, 39.9 ± 12.1 years), who showed no evidence of tumors during annual medical checkups (e.g., chest X‐ray and abdominal ultrasound for all volunteers as well as colonoscopy for individuals older than 40 years), were recruited to generate reference data. Written informed consent was obtained from all study participants before blood collection and genomic analysis of plasma cfDNA.

**Table 1:** Characteristics of healthy volunteers and patients

	Healthy volunteers (*n* = 50)	Patients with colorectal cancer (*n* = 10)	Patients with pancreatic cancer (*n* = 9)
Age (mean+/−SD)	21–63 (39.9 ± 12.1)	63–84 (72.4 ± 6.3)	62–81 (71.9 ± 7.1)
20–30 <	13	0	0
30–40 <	13	0	0
40–50 <	12	0	0
50–60 <	10	0	0
60–70 <	2	3	4
70–80 <	0	6	3
80 >	0	1	2
Gender (female : male)	24 : 26	4 : 6	6 : 3
Tumor stages (UICC; 0/I/II/III)	–	1/2/3/5	1/1/7/0

### 2.3 Plasma collection

Blood samples (limited to < 16 mL) were collected in 8‐mL tubes containing EDTA‐2K (SPM‐L1008EMS; Sekisui Medical, Co., Ltd., Tokyo, Japan) and gently inverted. Plasma was isolated within 2 h. Tubes were centrifuged at 1100 ***g*** for 10 min at 20–25 °C, followed by an additional centrifugation at 18 000 ***g*** for 10 min at 4 °C. The supernatant (cell‐free plasma) was collected in 2‐mL serum tubes and stored at −80 °C until analysis.

cfDNA was isolated from 2 mL plasma using a QIAamp Circulating Nucleic Acids Kit (Qiagen, Valencia, CA, USA) according to the manufacturer's instructions, eluted with 100 μL elution buffer, and immediately quantified using a Qubit dsDNA HS Assay Kit (Thermo Fisher Scientific, Waltham, MA, USA) and Qubit2.0 fluorometer (Thermo Fisher Scientific).

### 2.4 Cell lines

Hs766T and MIA PaCa‐2 cells were obtained from ATCC (Manassas, VA, USA) and Riken Bioresource Center (Tsukuba, Japan), respectively. Cells were grown at 37 °C in a 5% CO_2_ atmosphere in growth medium supplemented with 10% fetal bovine serum and 1% penicillin and streptomycin (Wako Pure Chemical Industries, Osaka, Japan). Cell culture supernatants were collected 24 h after medium replacement when the cell density reached 60–70% and cleared by two‐step centrifugation as described for blood samples. cfDNA was then isolated using a QIAamp Circulating Nucleic Acid Kit.

### 2.5 Mutation detection by digital pcr

Mutant *KRAS* variants (codons 12 and 13) in plasma cfDNA were analyzed using a QX200 Droplet Digital PCR System (Bio‐Rad, Hercules, CA, USA). Custom probes and primers were designed for eight major mutations in *KRAS* codons 12 and 13; sequences are presented in Table S1. The probes used in this study (Integrated DNA Technologies, Coralville, Iowa, USA) contained locked nucleic acid bases, which increased the binding specificity of the probes and enabled the detection of small mutant DNA fractions (Johnson *et al*., 2004). The sensitivity and specificity of the probes were initially validated using mutant *KRAS* genomic DNA (gDNA) cloned in the phrGFP‐N1 plasmid (Fig. S1).

Two sets of probes for four mutant *KRAS* genes in combination with the probe for the wild‐type *KRAS* were utilized to screen for mutant alleles. When the mutant *KRAS* was detected, an additional assay using the probe for a single mutation was performed to confirm the presence of a specific mutant allele.

The reaction mixture was prepared as described in Table S2. When plasma samples were analyzed, 9.3 μL of purified cfDNA (equivalent to cfDNA from 186 μL plasma) was used for a single PCR and partitioned into ~ 22 000 droplets per sample by mixing with 70 μL Droplet Generation Oil (Bio‐Rad) in a QX200 droplet generator (Bio‐Rad). Droplets were then subjected to thermal cycling using a Veriti Thermal Cycler (Thermo Fisher Scientific), as described in Table S3. Samples were transferred to a QX200 droplet reader (Bio‐Rad) for fluorescence measurement of 6‐fluorescein amidite (FAM) and hexachloro‐fluorescein (HEX) probes. Droplets were scored as positive or negative based on their fluorescence intensity, which was determined by the gating threshold defined using positive and negative controls. Finally, absolute copy number input in the reaction and the ratio of mutated fragment were calculated by quantasoft (ver 1.7; Bio‐Rad) based on the Poisson distribution. Plasma cfDNA samples were scored as positive for mutant *KRAS* when at least three mutant droplets/reaction were detected by ddPCR.

### 2.6 Preamplification of cfDNA

Preamplification of cfDNA was performed using the ddPCR platform. The reactions were prepared in a total volume of 22 μL containing 10 μL Master mix, forward and reverse primers (0.45 μm each), dNTP mixture (1.36 mm), and 5 μL purified cfDNA. The reaction mixture was emulsified as described above, and short‐cycle PCR was run to amplify the target allele (reaction conditions are described in Tables S3 and S4). The reaction mixtures were then diluted with TE buffer (pH 8.0), vigorously mixed with equal volumes of chloroform (Wako Pure Chemical Industries) by vortexing and pipetting, and centrifuged; the aqueous phase was separated, and the amplified DNA was then purified using a DNA Clean & Concentrator‐5 kit (Zymo Research, Irvine, CA, USA) according to the manufacturer's instructions. DNA was finally eluted using 12 μL of elution buffer and 4 μL of the purified products for subsequent second‐run ddPCR to detect mutations.

### 2.7 Mutation profiling of primary tumors

Primary tumor specimens were prepared as formalin‐fixed, paraffin‐embedded (FFPE) blocks and slides. gDNA was then purified and isolated from paired tumor and normal tissue samples using a GeneRead DNA FFPE Kit (Qiagen) according to the manufacturer's instructions.

Mutation profiles of primary tumors were determined by target amplicon sequencing using a next‐generation sequencer, as described previously (Imai *et al*., 2015). Ten to 40 ng gDNA was amplified by PCR using Ion AmpliSeq Cancer Hotspot Panel v2 (Thermo Fisher Scientific) containing 207 primer pairs for 50 oncogenes. Sequencing was performed using an Ion Personal Genome Machine System and Ion PGM 200 Sequencing Kit (Thermo Fisher Scientific) according to the manufacturer's instructions. Sequence reads were demultiplexed, quality‐filtered, and aligned to the human reference genome (GRCh37) using torrent suite software (ver. 5.0.4; Thermo Fisher Scientific). Variants were identified with variant caller software (ver. 5.0.4.0; Thermo Fisher Scientific).

### 2.8 Statistical analysis

Student's *t*‐tests were performed to determine differences in cfDNA concentrations and KRAS copy numbers. Linear regression analysis was performed to identify the relationships between cfDNA concentrations and *KRAS* copy numbers. The upper limit of the reference intervals was determined as the tentative cutoff value for positivity for mutant DNA and calculated as the mean + 1.96 standard deviation (SD), indicating the 97.5th percentile and estimated 95% confidence interval (Sunderman, 1975). Statistical analysis was performed using IBM spss Statistics Ver.22.0 (IBM, Armonk, NY, USA) and sas Studio Release 3.4 (Enterprise Edition; SAS Institute, Cary, NC, USA).

## 3 Results

### 3.1 Characteristics of cfDNA in patients with cancer and healthy individuals

In order to develop an appropriate protocol for plasma cfDNA genotyping, which can also identified patients with localized nonmetastatic cancer, we first evaluated the quality of cfDNA samples from curatively resected tumors (UICC Stages 0–III; Table 1). CRC and PDA were selected because a large proportion of these tumors harbor oncogenic *KRAS*, a key driver mutation in tumorigenesis.

Plasma was collected from 50 healthy volunteers, 10 patients with CRC, and nine patients with PDA (Table 1). Following DNA extraction from cell‐free plasma, cfDNA was quantified by fluorescence measurements.

**Figure 1:**
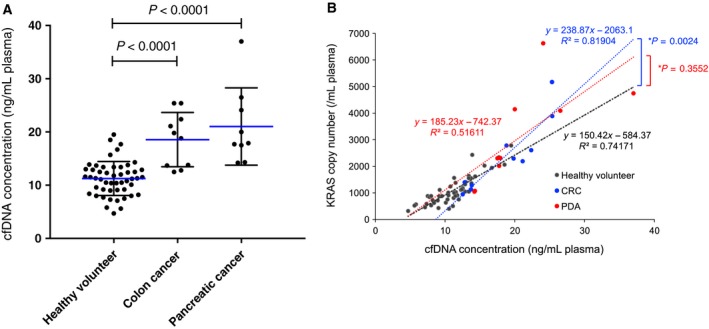
Characteristics of cfDNA extracted from plasma of patients with cancer and healthy volunteers. (A) Plasma cfDNA concentrations measured in 50 healthy volunteers, 10 patients with CRC, and nine patients with PDA; blue bars, mean cfDNA concentration (ng·mL^−1^ plasma). Bars indicate means (blue) and SDs (black). (B) Correlations between cfDNA concentrations and wild‐type *KRAS* copy numbers measured by ddPCR. Dots represent CRC (blue), PDA (red), and healthy individuals (gray). Linear regression analysis was performed to detect correlations; the regression equations and predicted *R* ^2^ values are shown.

Patients with cancer had higher cfDNA levels than healthy individuals (Fig. 1A); however, the difference was modest compared with that observed for advanced/metastatic diseases in previous studies (Takai *et al*., 2015). We next examined whether gene copy numbers measured by ddPCR correlated with cfDNA concentrations. Quantification of *KRAS* copy numbers using specific primer/probe sets (Table S1) revealed strong correlations between *KRAS* copy numbers and cfDNA concentrations in healthy individuals (*R* ^2 ^= 0.741; Fig. 1B). Such correlations were also observed in patients with CRC and PDA (*R* ^2 ^= 0.819 and *R* ^2^ = 0.516, respectively). Interestingly, patients with CRC showed a steeper slope of the regression line between *KRAS* copy numbers and cfDNA concentrations than healthy individuals (*P *=* *0.0024), whereas the difference between controls and patients with PDA was not significant (*P *=* *0.3552) (Fig. 1B).

### 3.2 ddPCR genotyping for mutant *kras* in patients with colorectal and pancreatic cancer

To test the sensitivity and specificity of mutant *KRAS* probes in the ddPCR genotyping assay, we performed preliminary experiments using cfDNA isolated from supernatants of cultured cells carrying a known *KRAS* mutation. DNA from *KRAS* ^*G12C*^ (MIA PaCa‐2; homozygous mutant) and wild‐type *KRAS* (Hs766T) cell lines was mixed at ratios of 1 : 10 (10%), 1 : 100 (1%), 1 : 1000 (0.1%), and 1 : 10 000 (0.01%) and amplified using probes for wild‐type *KRAS* (HEX) and mutant *KRAS* (FAM; multiplex pool#1 for *KRAS* ^*G12D*^, *KRAS* ^*G12V*^, *KRAS* ^*G12C*^, and *KRAS* ^*G13D*^; Tables S1 and S2). Detection of the mutant DNA in serially diluted samples showed a linear pattern, indicating that the detection limit in our assay was 0.01% (*R* ^2^ = 0.9950; Fig. S2).

**Figure 2:**
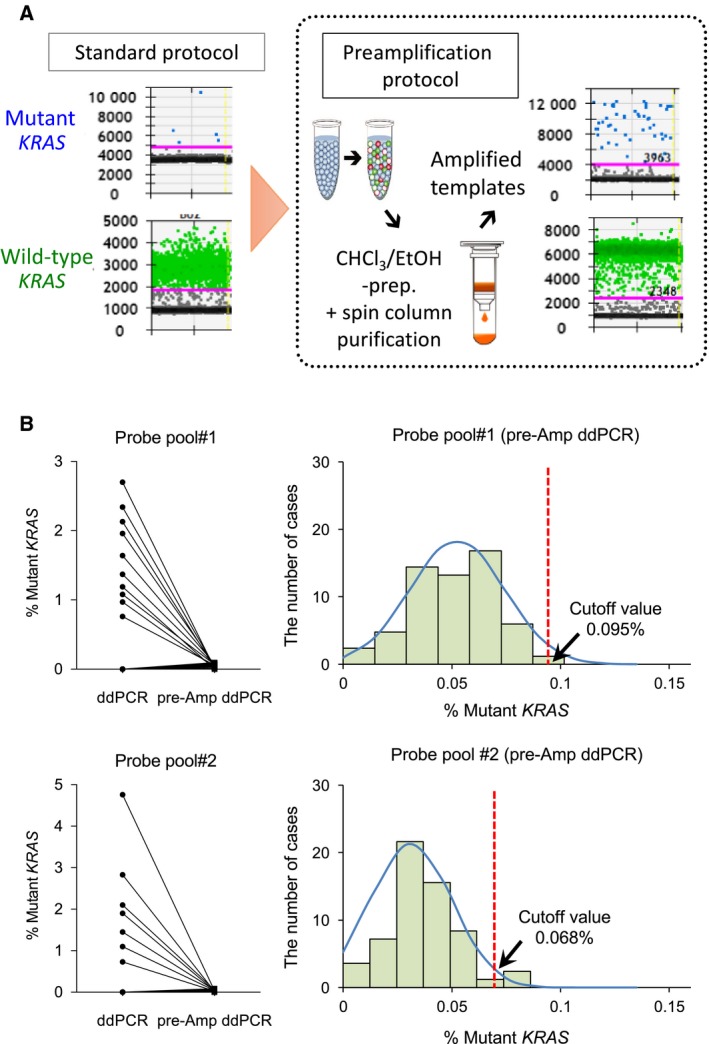
ddPCR‐based preamplification of cfDNA for *KRAS* genotyping. (A) Flow chart of preamplification in the ddPCR‐based assay. *KRAS* codon 12 and 13 alleles were preamplified from plasma cfDNA using the ddPCR platform, and the resulting fragments were purified. Mutation detection was then performed using second‐run ddPCR with a specific probe set. (B) Compared with the standard protocol, the modified method incorporating the preamplification step decreased PCR‐generated noise when cfDNA samples from 50 healthy volunteers were utilized (left panel). Given the normal probability distribution (blue line), the upper 97.5th percentile (mean + 1.96 SD; red dashed line) was taken as the cutoff value calculated as the mean and SD of the mutant *KRAS* frequency using *KRAS* probe pools #1 and #2 (right panel).

We next examined whether the ddPCR protocol could be used in practice. First, we utilized plasma cfDNA samples from healthy volunteers. In 10 and seven individuals, small numbers of mutant *KRAS* signals were called at more than 0.5% in frequency by probe sets #1 and #2, respectively (Fig. 2A). We then attempted the detection of mutant *KRAS* in cfDNA isolated from plasma of patients with CRC and PDA. Mutation screening by ddPCR resulted in the detection of mutant *KRAS* in five (35.7%) of 14 cases harboring mutant *KRAS* in primary tumors (Table 2). However, a significant fraction of mutant *KRAS* (over 0.5% allele frequency) was also detected in two patients with CRC with wild‐type *KRAS* (*n* = 5, 40%; Table 2).

**Table 2:** Results of plasma cfDNA genotyping

Case	Sex/age	Type of tumor	Stage (UICC)	Tumor volume (cm^2^)	cfDNA conc. (ng·mL^−1^)	*KRAS* in tumor	Standard protocol^a^			Preamplification protocol^b^		
							Mutant *KRAS* (copy/reaction)	WT *KRAS* (copy/reaction)	% ddPCR	Mutant *KRAS* (copy/reaction)	WT *KRAS* (copy/reaction)	% ddPCR with preamp.
1	F/78	CRC	IIIC	87.5	13.7	WT	1.47	210.2	0.70	6.6	12 070.7	0.05
2	F/64	CRC	IIIB	18.0	21.1	WT	1.54	407.0	0.38	19.8	28 842.0	0.07
3	M/68	CRC	IIA	600.0	25.4	WT	2.64	723.8	0.36	30.6	74 646.0	0.05
4	F/73	CRC	IIA	9.4	19.8	WT	1.83	426.6	0.43	17.6	41 250.0	0.04
5	M/84	CRC	I	3.2	18.8	WT	**6.88**	517.0	1.33	5.8	45 622.5	0.01
6	M/76	CRC	IIA	3.6	12.5	G13D	0.00	176.0	0	59.7	27 233.7	**0.22**
7	M/72	CRC	IIIB	36.0	25.4	G12V	**6.38**	961.4	0.66	31.7	94 908.0	0.03
8	M/72	CRC	0	24.5	22.4	G12D	2.42	484.0	0.50	16.5	56 034.0	0.03
9	F/74	CRC	IIIB	42.9	13.8	G13C	**9.02**	239.8	3.76	594.0	28 710.0	**2.07**
10	M/63	CRC	IIIB	50.6	12.8	G12V	**3.26**	260.7	1.25	24.4	22 391.1	**0.11**
11	M/79	PDA	IB	4.4	14.3	G12D	0.00	199.2	0	3.5	20 981.1	0.02
12	F/72	PDA	IIB	2.5	24.1	G12D	0.00	1232.7	0	244.2	78 210.0	**0.31**
13	F/65	PDA	IIA	1.8	14.2	G12V	**3.07**	194.4	1.58	23.1	24 618.0	**0.09**
14	F/66	PDA	IIB	1.3	17.9	G12R	0.00	429.7	0	47.5	31 482.0	**0.15**
15	F/62	PDA	0	0.1	20.0	G12D	0.00	770.7	0	41.4	35 376.0	**0.12**
16	M/81	PDA	IIA	0.8	17.5	G12R	**3.84**	428.4	0.90	0.0	52 470.0	0.00
17	M/81	PDA	IIA	81.3	26.5	G12D	0.00	761.6	0	34.2	114 057.8	0.03
18	F/72	PDA	IIA	7.1	17.7	G12R	2.05	375.1	0.55	16.7	53 548.0	0.03
19	F/69	PDA	IIB	10.9	37.0	G12D	1.61	882.9	0.18	145.2	66 660.0	**0.22**

a 9.3 μL template cfDNA was utilized and data are shown as copy/reaction (equivalent to 186 μL plasma) and frequency of the mutant allele. Boldface indicates positive sample as defined by more than three mutant copies/assay.

b 5 μL template cfDNA was utilized for first‐run preamplification. The second‐run ddPCR was performed using 30% volume of purified product of first‐run ddPCR. Data are shown as copy number/reaction (equivalent to 100 μL plasma) and frequency of the mutant allele. Boldface indicates positive sample as defined by mutant allele > 0.09%.

### 3.3 Requirement for cfDNA preamplification for detection of mutant cfDNA present in very low copy numbers in early‐stage cancer

As shown in Table 2, the copy number of the wild‐type *KRAS* isolated from 186 μL cell‐free plasma of patients with CRC or PDA was approximately ~ 1000/assay (509.5 ± 300.4). Given the low yield of cfDNA in plasma of patients with early‐stage cancer, most samples did not meet the capability of the sensitive platform. To overcome the potential subsampling issues and achieve better assay specificity, we next attempted preamplification of plasma cfDNA using primers flanking *KRAS* exon 2 as the first‐step PCR, which would generate the same amplicon as ddPCR used above.

DNA isolated from the supernatants of cultured cells carrying a known *KRAS* mutation was used to test the feasibility of the preamplification step for increasing template DNA. Test samples were prepared as described by mixing DNA from supernatants of *KRAS* mutant and wild‐type cells at ratios of 1 : 10 (10%) to 1 : 10 000 (0.01%). When the concentration of template DNA was very high (> 20 000 copies/assay), preamplification enabled the detection of ~ 0.01% mutant *KRAS*, which was consistent with the level achieved using the standard protocol (Fig. S3A). Rare mutant DNA could also be effectively amplified at up to 0.1% dilution (*R* ^2 ^= 0.9341), even when low‐yield DNA equivalent to the cfDNA concentration in plasma of patients with early‐stage cancer was utilized (around 4000 copies/assay; Fig. S3B). Eight cycles of the first‐step PCR were required to provide a minimum copy number (> 10) to capture 0.1% of mutant alleles (Fig. S4). Using maximally diluted template prepared from MIA PaCa‐2 supernatants in which the standard ddPCR protocol could rarely detect a few mutant *KRAS* copies, the ‘preamplification’ protocol indeed constantly provided positive signals for the mutation, resulting in dissolution of subsampling (Fig. S5; 10 errors in 16 reactions by the standard protocol and no error in 16 reactions by the modified protocol).

Next, we applied the modified ddPCR protocol to test 50 plasma samples from healthy individuals using eight cycles of preamplification (Fig. 2A,B, left panel). Given the low yield of plasma cfDNA from healthy volunteers, only a few copies (0–3.52 copies/reaction; mean 0.229 ± 0.558) of PCR‐generated errors caused a significantly high frequency of mutant alleles (over 0.5%) when the standard ddPCR protocol was used. In contrast, the modified protocol could substantially reduce noise. There were still a very small number of positive droplets captured by mutant probes after the modification, and the ratio of mutant to wild‐type *KRAS* in plasma cfDNA of healthy volunteers was dramatically decreased, following a normal distribution (Fig. 2A,B, right panel). The level was not associated with age (Fig. S6). The level of the cutoff limit for the assay was determined as 0.095% [confidence interval (CI): 0.084–0.105] for mutant *KRAS* probe pool #1 (*KRAS* ^*G12D*^, *KRAS* ^*G12V*^, *KRAS* ^*G12C*^, and *KRAS* ^*G13D*^) and 0.068% (CI: 0.059–0.077) for pool #2 (*KRAS* ^*G12R*^, *KRAS* ^*G12A*^, *KRAS* ^*G12S*^, and *KRAS* ^*G13C*^; Fig. 2A,B, right panel, and Table S5). The value was slightly higher than the detection limit of the ddPCR protocol.

**Figure 3:**
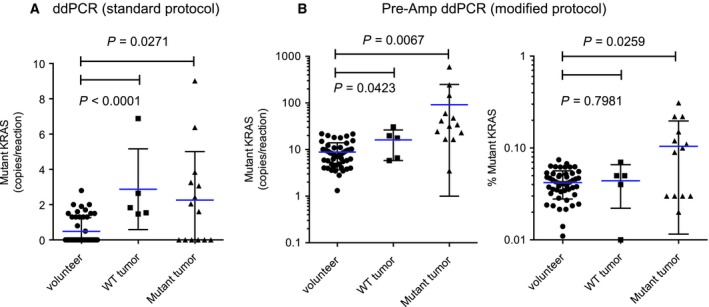
Increased specificity of ddPCR‐based *KRAS* genotyping assay by preamplification. (A) Using a low yield of plasma cfDNA, a few copies of PCR‐generated noise using the standard ddPCR protocol masked the difference between *KRAS* wild‐type and mutant samples. (B) The preamplification procedure overcame the subsampling issue and significantly improved the signal‐to‐noise ratio. Copy numbers for mutant *KRAS* (left panel) and the frequency (right panel) are shown. Bars indicate means (blue) and SDs (black).

Finally, we determined whether the preamplification step could indeed enhance the detection sensitivity for rare variants in plasma cfDNA of patients with cancer. Preamplification was performed using 5 μL cfDNA (equivalent to 100 μL plasma, containing 1.97 ± 0.61 ng DNA), and approximately 30% of the amplified template was utilized for the second‐run ddPCR assay, resulting in successful detection of the *KRAS* mutation in 8 (57.1%) of 14 *KRAS* mutant tumors (five CRC and nine PDA) samples (Table 2, Fig. 3, and Table S6). In the majority of cases, concordance of the mutation between the primary tumor and cfDNA was verified using a single probe that matched the mutation present in the tumor at the nucleotide level using the modified protocol. In addition, false‐positive signals for mutant KRAS obtained in healthy volunteers and patients with wild‐type *KRAS* tumors by the standard protocol were also substantially eliminated by the preamplification step (Fig. 3B).

## 4 Discussion

cfDNA shed from tumors into the general circulation has been studied to monitor tumor genetics and may be used in a sensitive and minimally invasive method for systematic tracing of cancer genomic evolution (Crowley *et al*., 2013; Forshew *et al*., 2012). Recent technological advances in genetics have allowed ultrasensitive and absolute quantification of very low‐abundance mutant alleles, even in highly diluted specimens, such as blood and urine (Didelot *et al*., 2013; Hyman *et al*., 2015). Among such methodologies, the digital PCR platform is an established tool for genotyping, but is still far from being a comprehensive assay. Therefore, in this study, we examined its feasibility as a clinical test for the diagnosis of early rather than advanced cancer stages.

Although plasma cfDNA concentrations were generally high in patients with advanced cancer (Schwarzenbach *et al*., 2011), the yield in patients with early‐stage tumors is not significantly different from that in healthy individuals. Indeed, in the current study, the average cfDNA concentration in patients with curatively resectable CRC or PDA did not exceed much more than 20 μg·L^−1^ plasma. DNA fragments in plasma from patients with CRC have been shown to be longer than those in healthy subjects, allowing the simple detection of mutant DNA (Heitzer *et al*., 2013); however, other studies have shown that the mutant allele occurs more commonly at a shorter fragment length (Underhill *et al*., 2016), and the size can vary and is highly dependent on the type of tissue from which the cfDNA is released (Jiang *et al*., 2015; Snyder *et al*., 2016). We compared *KRAS* copy numbers in patients with early‐stage cancer and healthy individuals; surprisingly, DNA fragments captured by standard ddPCR in patients with PDA were not larger than those from healthy controls when normalized by plasma cfDNA concentrations. The absolute value was around ~ 5000 copies·mL^−1^ plasma when standard blood collection and cfDNA purification methods were used. Considering the volume of blood collection tubes (~ 10 mL), it is not feasible to detect more than 20 000 copies of target alleles for several driver genes using the standard ddPCR diagnostic protocol. Therefore, available plasma cfDNA from patients with curatively resectable tumors is not sufficient for ddPCR‐based quantification of very rare mutant alleles.

Indeed, mutation assays of the *KRAS* gene using standard ddPCR protocols provided a very low concordance between plasma and tissue and concerns regarding specificity, particularly in curatively resectable PDAs. This was not surprising because limited amounts of cfDNA could be used; additionally, these results could be explained, in part, by subsampling issue (Lievens *et al*., 2016). Moreover, a previous report demonstrated that there were large variations in the frequencies of mutant alleles present in plasma and that the average levels in PDA were 10–100 times lower than those in CRC (Bettegowda *et al*., 2014). Given low neoplastic cellularity of pancreatic cancers, it is more challenging to capture cfDNA derived from PDA than that from CRC, which is characterized by significantly higher tumor cellularity, that is, the relative proportion of tumor to normal cells in a sample. Such histological characteristics, as well as the rate of tumor cell turnover, may affect the sensitivity of cfDNA genotyping. In addition, limited yields of cfDNA may also decrease the specificity of mutation detection as a very small amount of PCR‐generated errors can cause considerable noise, even in samples from healthy individuals.

To circumvent the limitations posed by the low yields of template DNA for clinical sequencing, we decided to simply amplify target alleles with limited representation in patient cfDNA pools rather than collecting larger blood volumes. The first‐run ddPCR for preamplification and subsequent ddPCR‐based detection assays for mutant and wild‐type *KRAS* are likely to meet the requirements for practical use. The modified ddPCR protocol based on the amplification of a target allele enabled genotyping of over 10 000 copies from 100 μL plasma, indicating that its sensitivity finally met the requirement for the detection of rare mutations (~ 0.1%) in small blood volumes. More importantly, the subsampling issue observed by standard ddPCR was dramatically attenuated by preamplification, allowing us to capture mutant alleles at a very low abundance.

Furthermore, using the modified protocol, the impact of minute PCR‐generated errors could be sufficiently cancelled, resulting in a dramatic noise reduction. This procedural modification conferred the ddPCR platform with high specificity for minimally invasive diagnostic tests. Careful assessment is required to determine the cutoff for abnormal levels because this method cannot fully overcome the issue of PCR noise. Here, we determined the provisional cutoff line as ~ 0.1% based on statistical analysis of the data from 50 healthy individuals when multiplex probe cocktails were used (0.068–0.095%). We could not completely exclude the possibility that a very rare mutant *KRAS* allele detected in the volunteers originated from latent precursor lesion(s) of tumors harboring oncogenic *KRAS* (Fernandez‐Cuesta *et al*., 2016; Gormally *et al*., 2006). Therefore, the cutoff level should be determined based on a larger number of volunteers and long‐term surveillance. Notably, the average allele frequency for mutant *KRAS* was not associated with the age of the volunteers; this should be re‐evaluated using samples from elderly individuals.

The major limitation of the current study was the small number of patients with cancer recruited for the modified protocol. A larger‐scale study is required to determine the abnormal range of mutation frequencies measured by the ddPCR assay incorporating a preamplification step, and we have an ongoing study to evaluate the feasibility of the assay (UMIN000012810). In addition, a long‐term surveillance study is necessary to conclude whether liquid biopsy material analyzed using the current protocol is suitable for risk stratification of patients with premalignant diseases, such as pancreatic cysts, and individuals with a strong family history of cancer (Gala *et al*., 2014; Tada *et al*., 2006; Vasen *et al*., 2016). Further optimization of the preamplification step may be achieved using multiplex primer sets to simultaneously detect major driver mutations. Improvement of blood sampling and shipping processes by cfDNA stabilization and standardized quality control would promote practical use of such an assay (Malentacchi *et al*., 2015; Norton *et al*., 2013).

## 5 Conclusions

The major technical challenge for widespread implementation of cfDNA genotyping is insufficient detection sensitivity in cases of early‐stage cancers when the amount of cfDNA is very limited. Introduction of a preamplification step improved the analytic potential of the highly specific ddPCR‐based assay, establishing a reliable framework for digital quantification of oncogenic *KRAS* variants and other driver mutations for cancer screening.

## Author contributions

YO and YM conceived and designed the study and wrote the initial draft of the manuscript. YO performed most of experiments, with assistance from MO, RN, and TD. YO and KH performed genetic analysis and interpreted the data. AS and JS performed statistical analysis. HK, TY, SA, KK, and KA collected, analyzed, and interpreted clinical data. YM contributed to critical revision of the manuscript for important intellectual content. YO and YM obtained funding. The final version of the manuscript was approved by all authors.

## Supporting information


**Fig. S1.** Sensitivity and specificity of the *KRAS* probes containing locked nucleic acid (LNA) bases.
**Fig. S2.** ddPCR‐based detection of *KRAS* codons 12/13 by serial dilution.
**Fig. S3.** Serial dilution of pre‐amplified template alleles for *KRAS* genotyping assays.
**Fig. S4.** Validation of the number of PCR cycles in the pre‐amplification step.
**Fig. S5.** Improvement of the stochastic subsampling issue by pre‐amplification.
**Fig. S6.** Correlation between the ages of healthy volunteers and the frequency of mutant *KRAS* detected by ddPCR with pre‐amplification.

**Table S1.** Sequence of primers and probes.

**Table S2.** Preparation of ddPCR reaction mix.

**Table S3.** PCR protocol.

**Table S4.** Preparation of pre‐amplification reaction.

**Table S5.** Descriptive statistics of the ratio of mutant KRAS versus wild‐type KRAS fragments in plasma cfDNA.

**Table S6.** Sensitivity and specificity of the two ddCPR protocols.
