# Supplementary material for: An improved digital polymerase chain reaction protocol to capture low‐copy KRAS mutations in plasma cell‐free DNA by resolving ‘subsampling’ issues
Source: Mol Oncol. 2017 Aug 8;11(10):1448–58. doi: 10.1002/1878-0261.12110 (PMC5623814; doi:10.1002/1878-0261.12110)
Supplement: Supplementary file 1 — Fig. S1. Sensitivity and specificity of the KRAS probes containing locked nucleic acid (LNA) bases. Fig. S2. ddPCR‐based detection of KRAS codons 12/13 by serial dilution. Fig. S3. Serial dilution of pre‐amplified template alleles for KRAS genotyping assays. Fig. S4. Validation of the number of PCR cycles in the pre‐amplification step. Fig. S5. Improvement of the stochastic subsampling issue by pre‐amplification. Fig. S6. Correlation between the ages of healthy volunteers and the frequency of mutant KRAS detected by ddPCR with pre‐amplification. [file MOL2-11-1448-s001.pptx]

## Slide 1
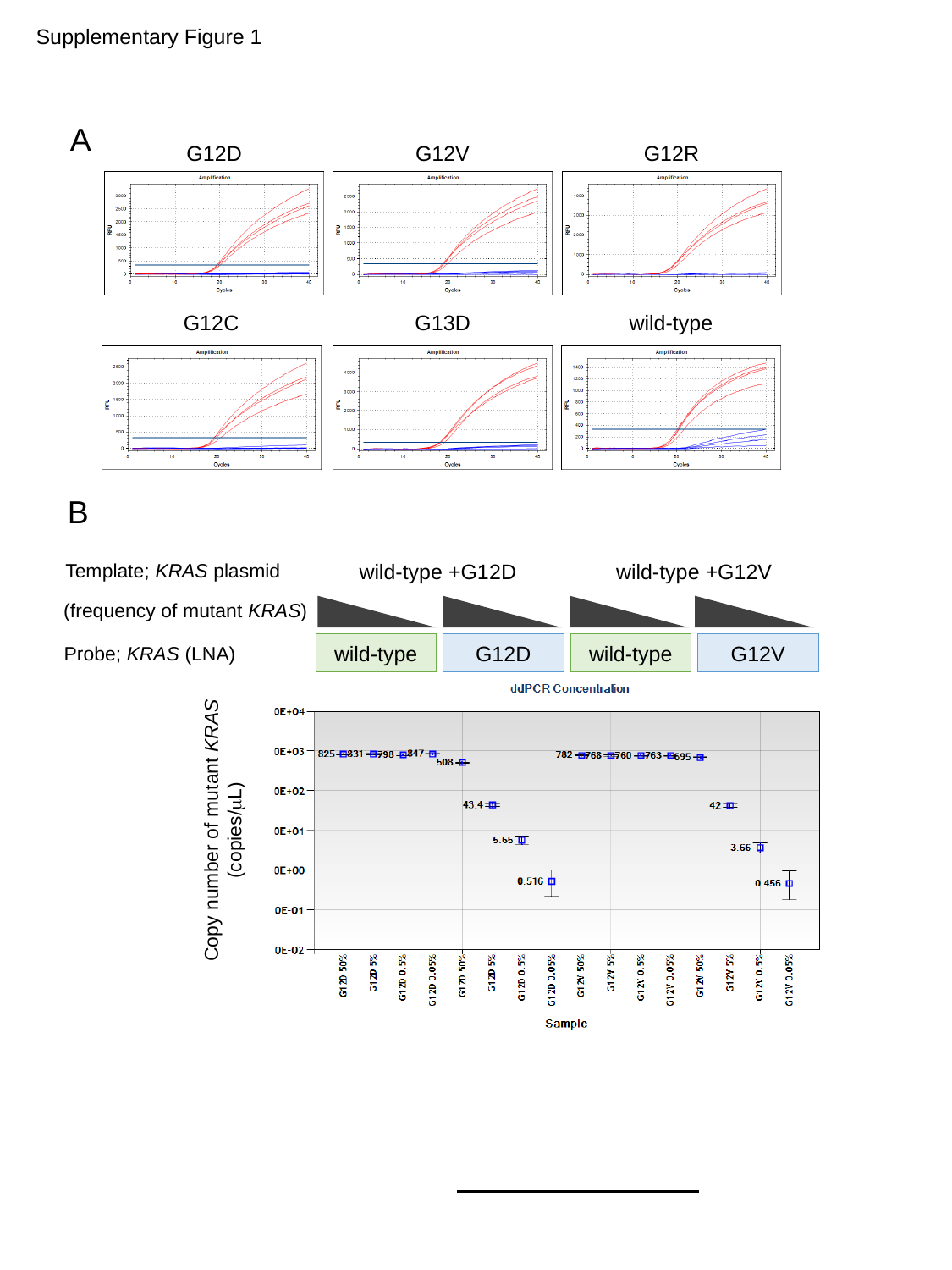

Supplementary Figure 1
A
G12D
G12V
G12R
G12C
G13D
wild-type
B
Template; KRAS plasmid
wild-type +G12D
wild-type +G12V
(frequency of mutant KRAS)
wild-type
G12D
wild-type
G12V
Probe; KRAS (LNA)
Copy number of mutant KRAS
(copies/mL)

## Slide 2
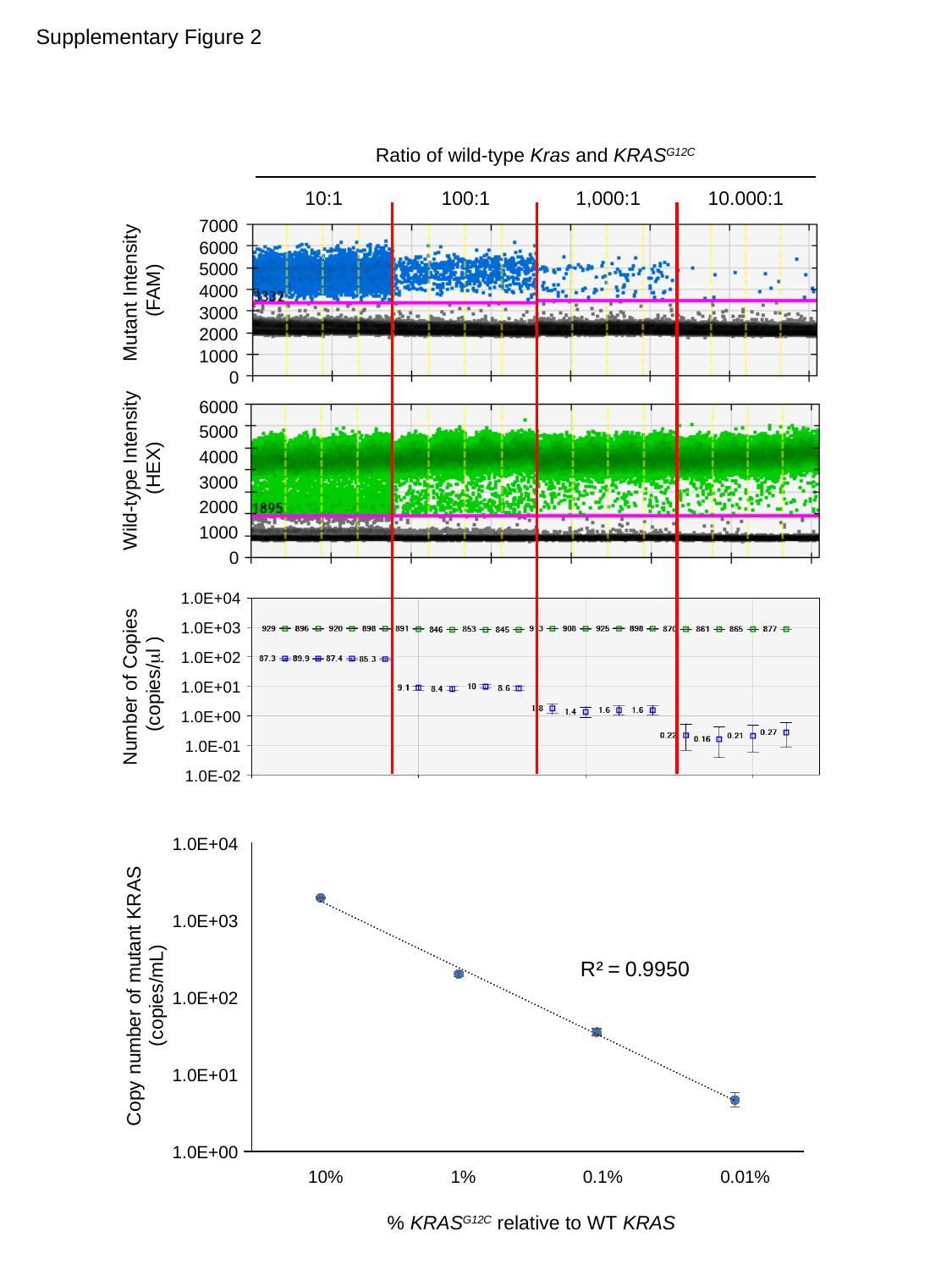

Supplementary Figure 2
Ratio of wild-type Kras and KRASG12C
10:1
100:1
1,000:1
10.000:1
7000
6000
5000
Mutant Intensity
 (FAM)
4000
3000
2000
1000
0
6000
5000
4000
Wild-type Intensity
 (HEX)
3000
2000
1000
0
1.0E+04
1.0E+03
1.0E+02
Number of Copies
 (copies/ml )
1.0E+01
1.0E+00
1.0E-01
1.0E-02
### Chart
| Category | | | | | | | | |
|---|---|---|---|---|---|---|---|---|1.0E+04
1.0E+03
1.0E+02
1.0E+01
1.0E+00
10%
1%
0.1%
0.01%
% KRASG12C relative to WT KRAS

## Slide 3
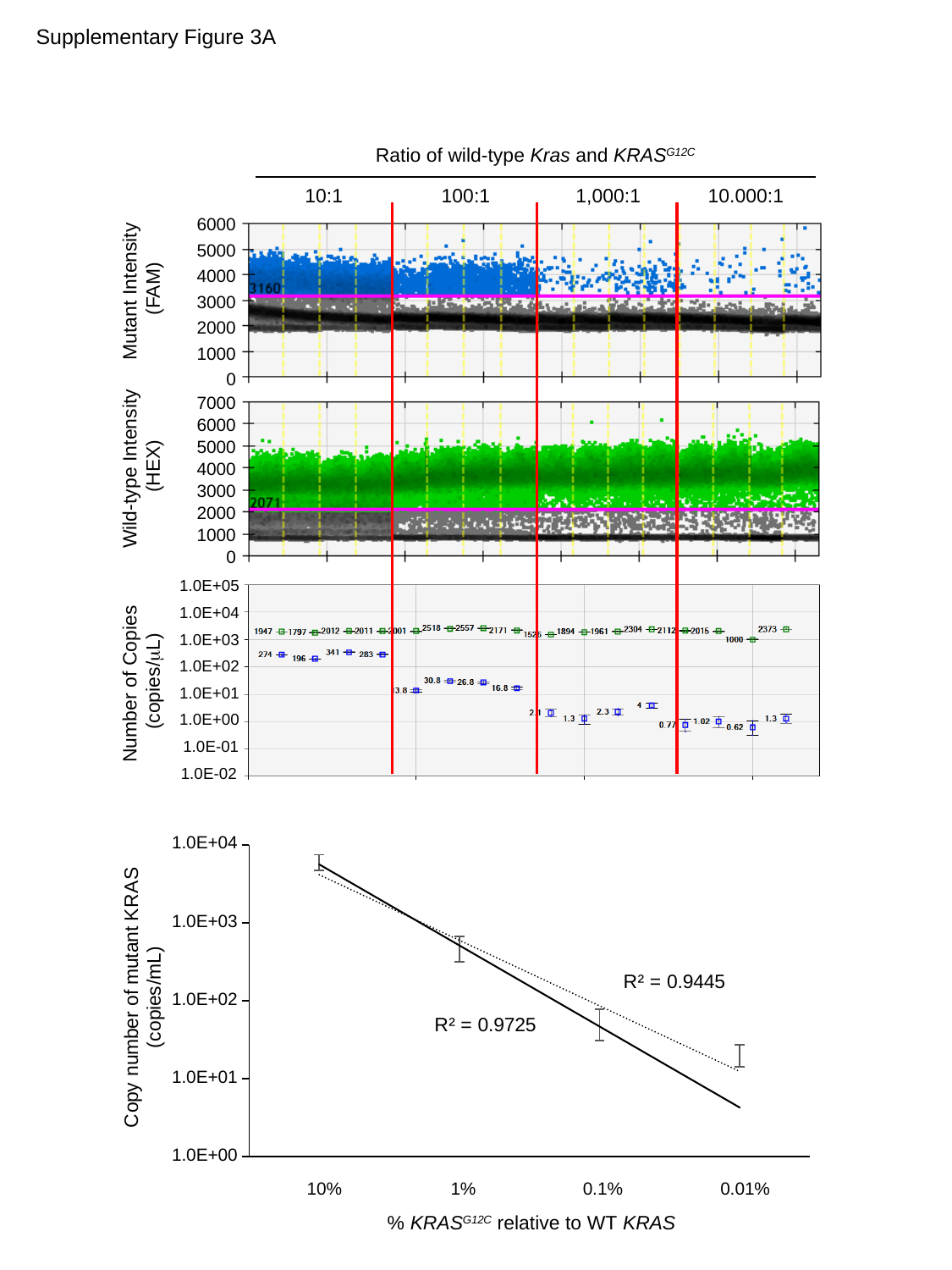

Supplementary Figure 3A
Ratio of wild-type Kras and KRASG12C
10:1
100:1
1,000:1
10.000:1
6000
5000
4000
Mutant Intensity
 (FAM)
3000
2000
1000
0
7000
6000
5000
Wild-type Intensity
 (HEX)
4000
3000
2000
1000
0
1.0E+05
1.0E+04
1.0E+03
1.0E+02
Number of Copies
 (copies/mL)
1.0E+01
1.0E+00
1.0E-01
1.0E-02
### Chart
| Category | | |
|---|---|---|1.0E+04
1.0E+03
1.0E+02
1.0E+01
1.0E+00
10%
1%
0.1%
0.01%
% KRASG12C relative to WT KRAS

## Slide 4
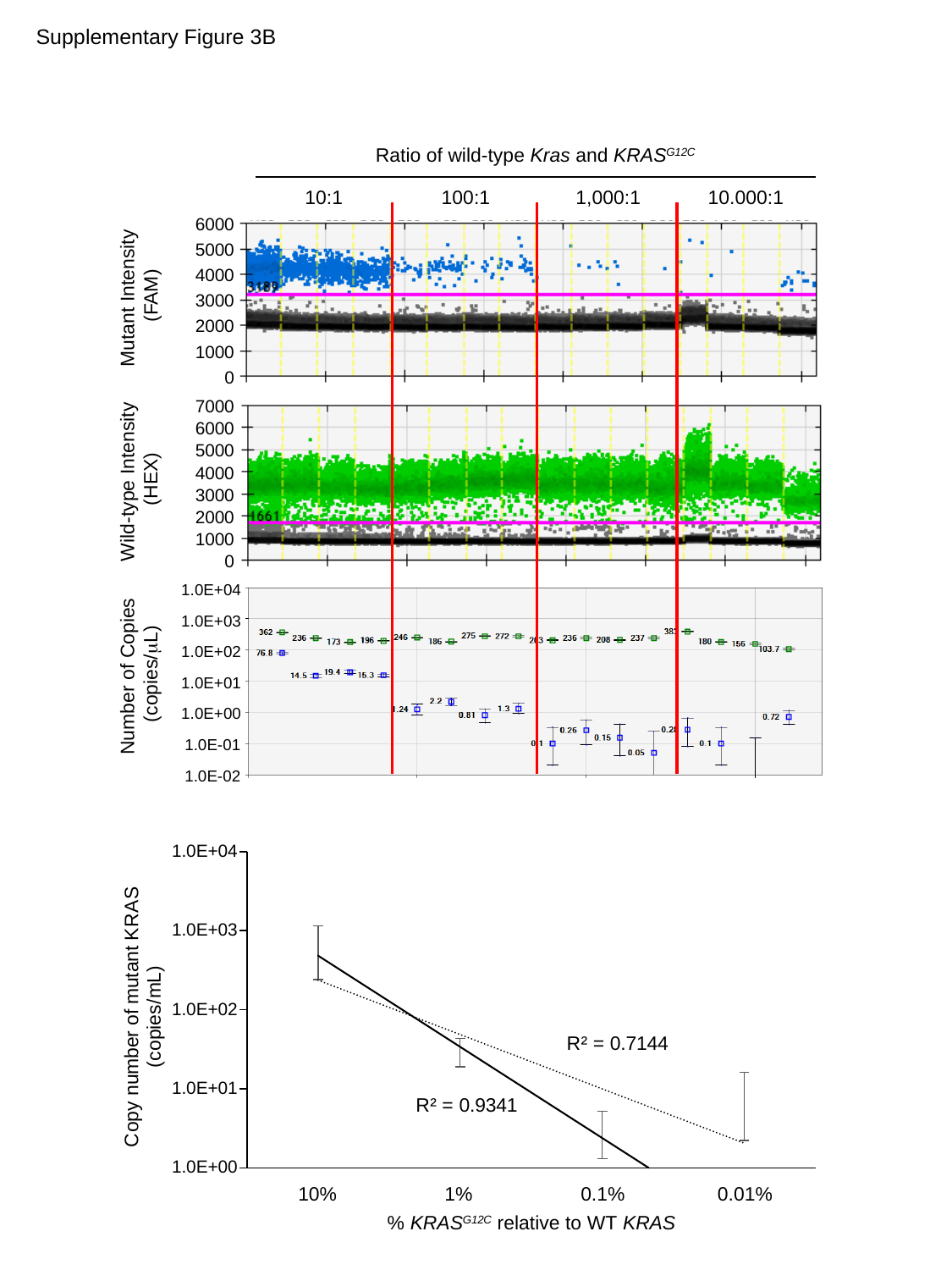

Supplementary Figure 3B
Ratio of wild-type Kras and KRASG12C
10:1
100:1
1,000:1
10.000:1
6000
5000
4000
Mutant Intensity
 (FAM)
3000
2000
1000
0
7000
6000
5000
Wild-type Intensity
 (HEX)
4000
3000
2000
1000
0
1.0E+04
1.0E+03
1.0E+02
Number of Copies
 (copies/mL)
1.0E+01
1.0E+00
1.0E-01
1.0E-02
### Chart
| Category | | |
|---|---|---|1.0E+04
1.0E+03
1.0E+02
1.0E+01
1.0E+00
10%
1%
0.1%
0.01%
% KRASG12C relative to WT KRAS

## Slide 5
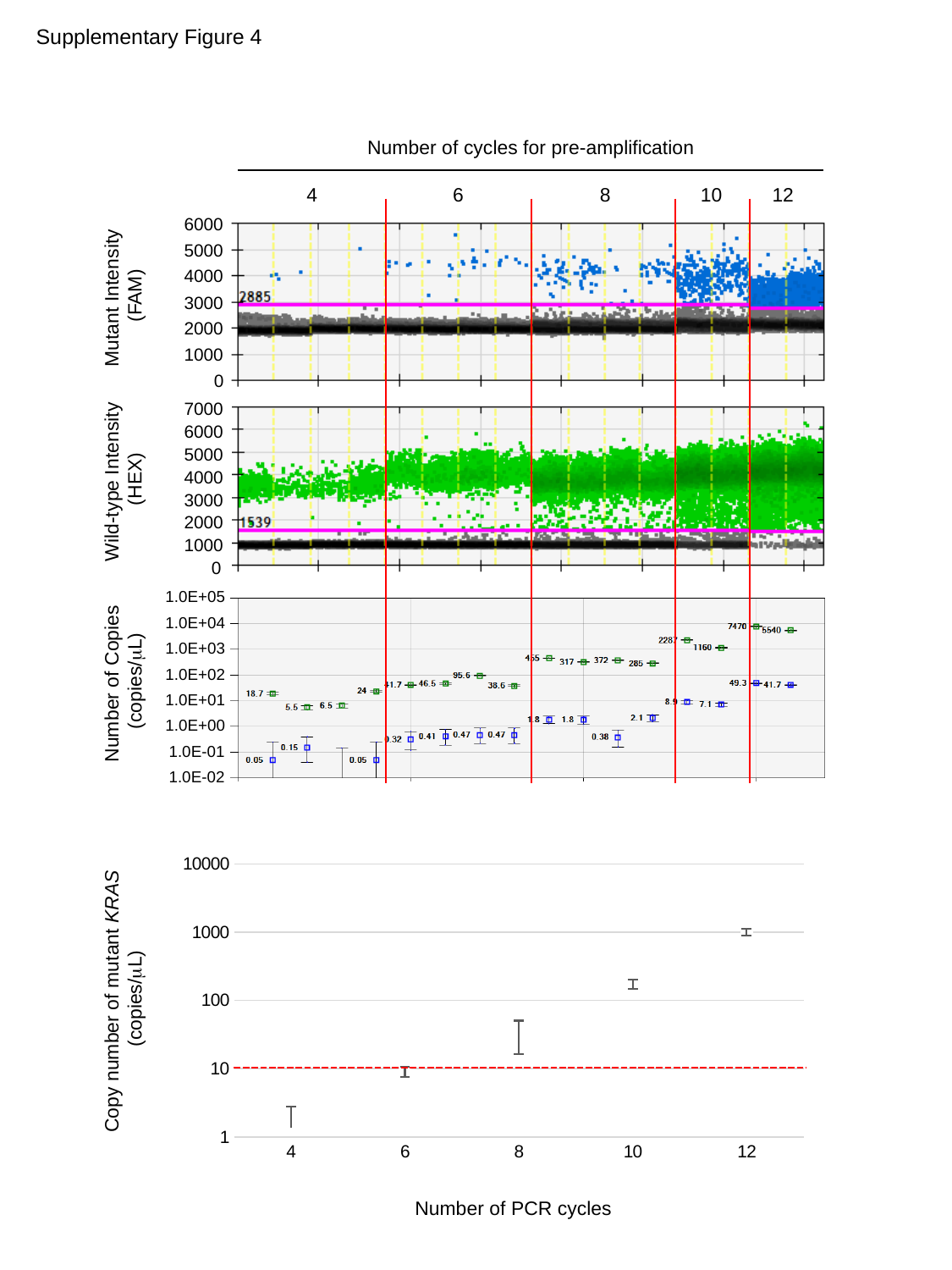

Supplementary Figure 4
Number of cycles for pre-amplification
4
6
8
10
12
6000
5000
4000
Mutant Intensity
 (FAM)
3000
2000
1000
0
7000
6000
5000
Wild-type Intensity
 (HEX)
4000
3000
2000
1000
0
1.0E+05
1.0E+04
1.0E+03
Number of Copies
 (copies/mL)
1.0E+02
1.0E+01
1.0E+00
1.0E-01
1.0E-02
### Chart
| Category | 平均 |
|---|---|
| 4.0 | 1.375 |
| 6.0 | 9.185 |
| 8.0 | 33.44 |
| 10.0 | 176.0 |
| 12.0 | 1001.0 |Copy number of mutant KRAS
 (copies/mL)
Number of PCR cycles

## Slide 6
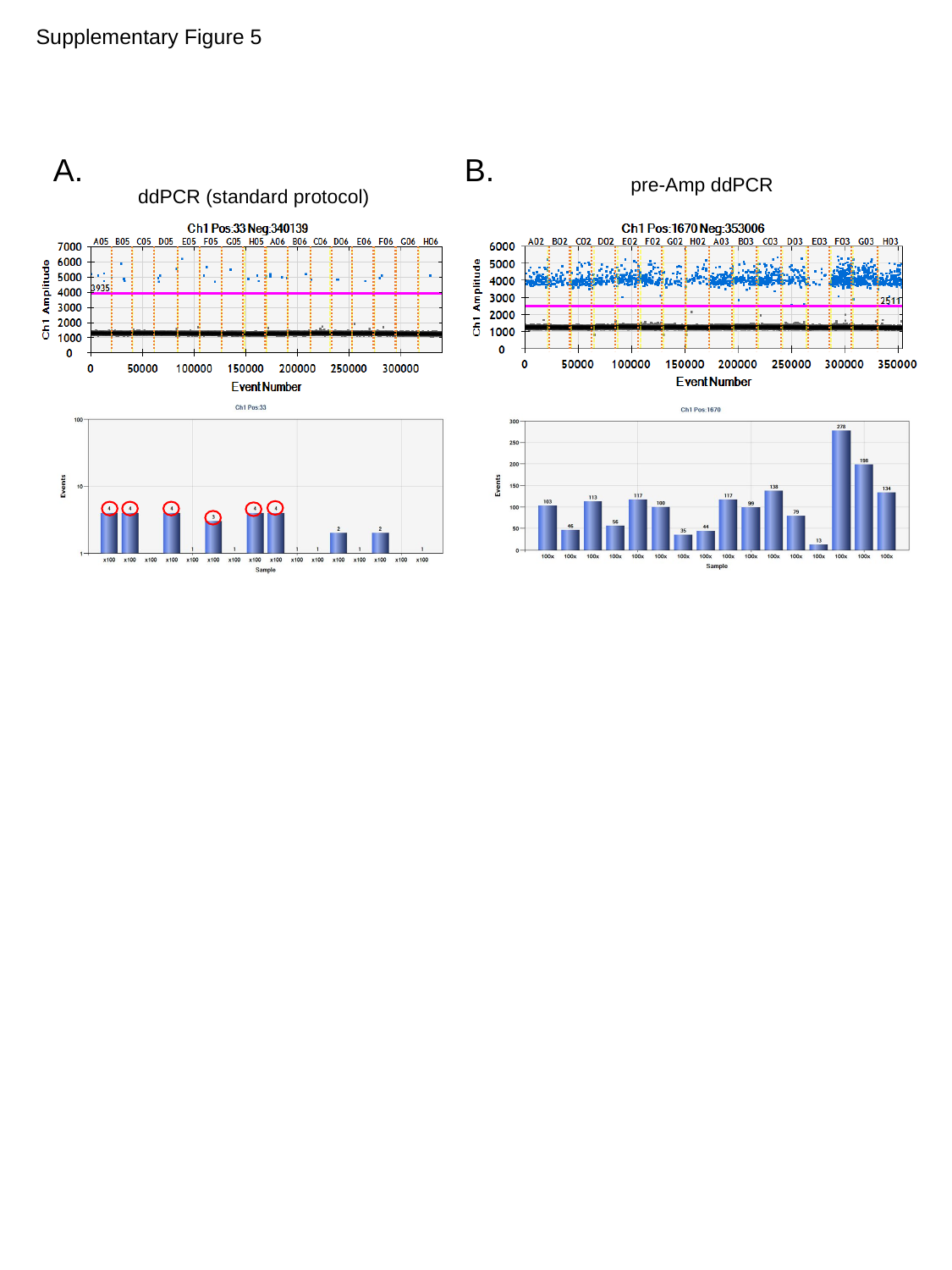

Supplementary Figure 5
A.
B.
pre-Amp ddPCR
ddPCR (standard protocol)

## Slide 7
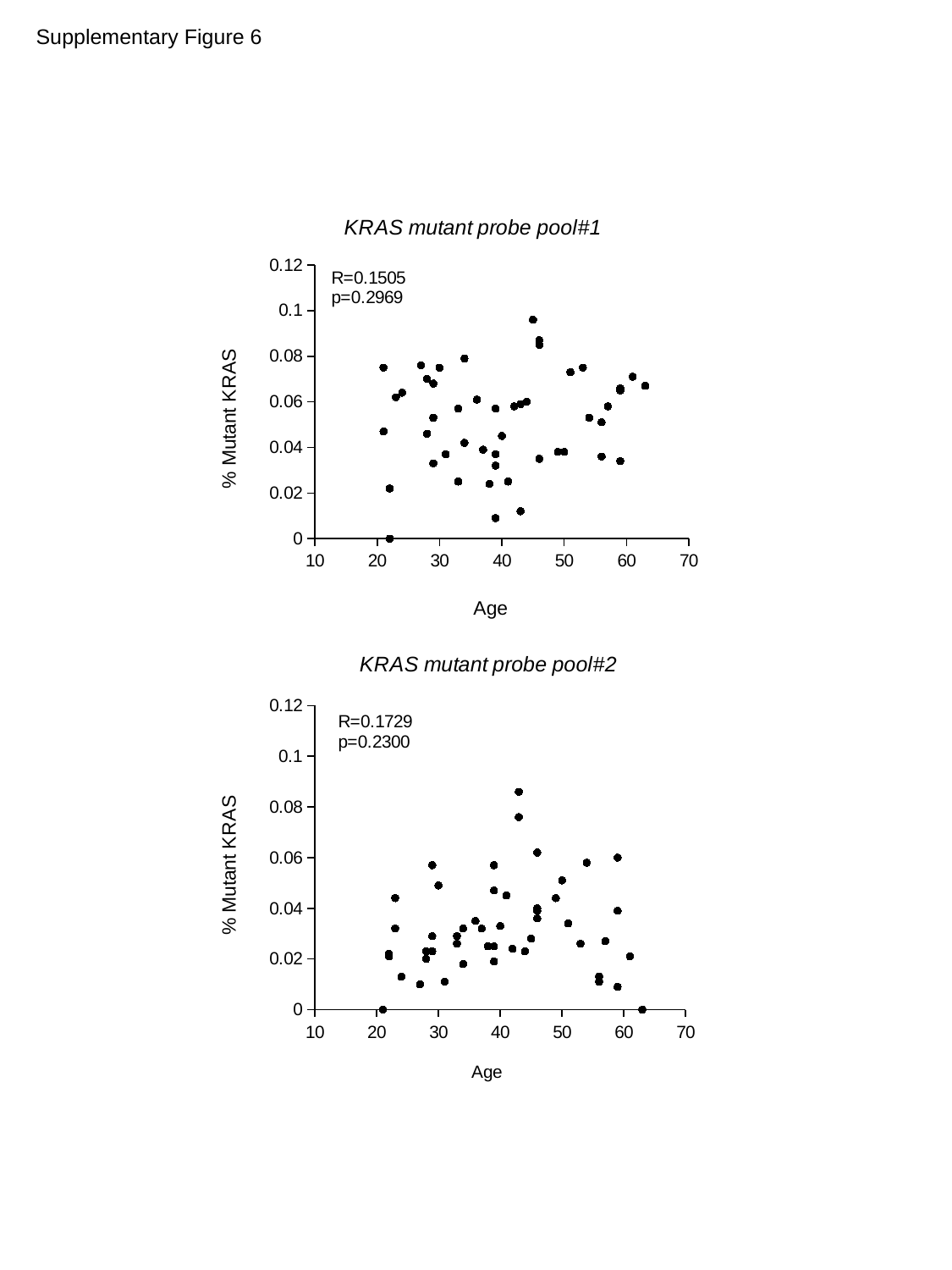

Supplementary Figure 6
### Chart: KRAS mutant probe pool#1
| Category | P1PA_pct_Mut |
|---|---|
### Chart: KRAS mutant probe pool#2
| Category | P2PA_pct_Mut |
|---|---|
